# A programme to increase appropriate usage of benzathine penicillin for management of streptococcal pharyngitis and rheumatic heart disease in Zambia

**DOI:** 10.5830/CVJA-2017-002

**Published:** 2017

**Authors:** Aidan Long, Joyce Chipili Lungu, Elizabeth Machila, John Musuku, Sherri Schwaninger, Jonathan Spector, Brigitta Tadmor, Mark Fishman, Bongani M Mayosi

**Affiliations:** Massachusetts General Hospital, Boston, USA; University Teaching Hospital, Lusaka, Zambia; University Teaching Hospital, Lusaka, Zambia; University Teaching Hospital, Lusaka, Zambia; Novartis Institutes for BioMedical Research, Cambridge, USA; Novartis Institutes for BioMedical Research, Cambridge, USA; Novartis Institutes for BioMedical Research, Cambridge, USA; Harvard Stem Cell and Regenerative Biology Department, Harvard University, Cambridge, USA; Department of Medicine, Groote Schuur Hospital and University of Cape Town, Cape Town, South Africa

**Keywords:** rheumatic fever, rheumatic heart disease, benzathine penicillin, pencillin allergy

## Abstract

Rheumatic heart disease is highly prevalent and associated with substantial morbidity and mortality in many resourcepoor areas of the world, including sub-Saharan Africa.Primary and secondary prophylaxis with penicillin has beenshown to significantly improve outcomes and is recognisedto be the standard of care, with intra-muscular benzathine penicillin G recommended as the preferred agent by many technical experts. However, ensuring compliance with therapyhas proven to be challenging. As part of a public–privatepartnership initiative in Zambia, we conducted an educationaland access-to-medicine programme aimed at increasing appropriate use of benzathine penicillin for the preventionand management of rheumatic heart disease, according tonational guidelines. The programme was informed early onby identification of potential barriers to the administration ofinjectable penicillin, which included concern by health workers about allergic events. We describe this programme andreport initial signs of success, as indicated by increased useof benzathine penicillin. We propose that a similar approach may have benefits in rheumatic heart disease programmes in other endemic regions.

## Introduction

Rheumatic heart disease (RHD) is a major cause of morbidity and mortality in sub-Saharan Africa (SSA).^1^,^2^ Up to 3% of school-aged children have definite or borderline RHD,^3^-^5^ and congestive heart failure stemming from valve damage in RHD patients is a leading non-infectious cause of death in young adults.^6^,^7^ Acute heart failure from RHD in SSA has been associated with a 35% one-year mortality rate.^8^

Yet RHD is preventable and, to some degree, treatable. Evidence generated more than 60 years ago demonstrated that antibiotic treatment of group A Streptococcus (GAS) pharyngitis, a practice known as ‘primary prevention’, significantly reduced the risk of rheumatic fever (RF).^9^-^11^ Shortly thereafter, it was shown that ‘secondary prevention’, in which antibiotics are administered continuously for a period of many years to patients with RHD, was effective at suppressing new streptococcal infections and decreased the incidence of recurrent RF.^10^,^12^-^14^ The initial RF and RHD studies used penicillin as the antibiotic of choice and, to this day, GAS remains exquisitely sensitive to penicillin treatment.^15^-^17^ Penicillin continues to be the standard of care for primary and secondary prevention of RHD globally in non-allergic individuals.^2^

In resource-constrained parts of the world where RHD is still endemic, including SSA, the use of penicillin for RHD prevention and treatment is widely recognised to be suboptimal.^18^-^20^ The reasons for this are complex and related to a multitude of interacting factors, including drug supply, pharmaco-economics, health service infrastructure and possibly socio-cultural drivers.^21^ Indeed, a recent high-level report outlining the key actions required to eradicate RHD in Africa identified variable supply and suboptimal quality and use of penicillin as some of the major barriers to achievement of this goal,^22^ a position endorsed by the World Heart Federation.^23^

Penicillin comes in various formulations. Benzathine penicillin G (BPG), a World Health Organisation essential medicine, is anintramuscular injectable form with a long half-life, such that only a single dose is required for primary prevention (in contrast to a 10-day course of oral pills taken twice daily), and a single monthly dose is needed for secondary prevention (compared with a regimen of oral pills taken twice daily).^10^

In SSA, leading technical authorities, including the Pan-African Society of Cardiology (PASCAR), have advocated the use of BPG for the treatment of streptococcal pharyngitis and the management of RHD to maximise the likelihood of patient compliance with recommended regimens, an approach that has met with success in other low-resource settings.^22^,^24^,^25^ There is also evidence that BPG may be more effective than oral penicillin for secondary prophylaxis of RHD and, consequently, it is a commonly recommended therapy.^14^,^26^,^27^

In 2012, a public–private partnership was launched in Zambia with the goal of reducing and ultimately eliminating RHD.^28^ This multi-faceted initiative (called ‘BeatRHD Zambia’) is centred outof the University Teaching Hospital (UTH) in Lusaka, Zambia,and includes operational research (for example, to measure diseaseprevalence), public awareness, and health system-strengtheningactivities – in particular, efforts to increase appropriate BPG usage for primary and secondary prevention of RHD in government health facilities according to national guidelines.

To explore and address factors contributing to possible low rates of BPG use among health workers in Zambia, we undertookan assessment of health workers’ attitudes and practices relating to BPG safety, appropriate use and effectiveness. The information obtained was used to inform education and training, andinterventions for BPG access, which have been implemented inhealth centres across Lusaka, Zambia, and are now being rolledout in other provinces. This report describes the experience todate of supporting the use of BPG for primary and secondary prevention of RHD in Zambia.

## Unmasking potential barriers to penicillin administration

A two-day workshop was conducted at UTH in October 2014 in order to elicit participants’ knowledge, attitudes and practicesrelating to RHD and BPG, and to provide education andtraining on how to administer BPG. The workshop involved aclassroom-based didactic and interactive programme directed atrepresentatives from UTH and 20 government clinics in Lusaka.There were 29 attendees, mostly nurses and a few doctors.

## Focus group discussion

An initial focus group discussion (led by AL and JM) permitted course leaders to gain insight into current patterns of penicillin usage in cases of streptococcal pharyngitis and RHD. It allowedfor an informal exploration of factors that were perceived to limitthe use of BPG in these clinical circumstances. All 29 workshopparticipants expressed awareness of the existence of RHD andthe majority reported having been involved in the care of such patients. While most participants reported prior experience with administration of oral penicillin VK and intramuscular penicillin G, no participant was able to relate first-hand experience in theadministration of intramuscular BPG.

Precise identification of the reasons for the non-use of BPG was challenging to ascertain but one theme appeared central: fear ofpenicillin allergy as a potential barrier to administration of BPGin Zambia. This concern had also been brought to light before theworkshop by personal interactions between Zambian nurses anddoctors and the head of Paediatrics at UTH (JM), which revealedanxiety over a perceived high risk of penicillin allergy associatedwith injectable penicillin (distinct from the oral form of penicillin).

During the focus group, a significant number of the participants expressed grave fear of inducing an allergic reaction, apparently based on anecdotal information they had received secondhand about such events. No participant reporteddirectly having encountered an adverse drug reaction (includingallergic or anaphylactic reactions) with administration of anyformulation of penicillin. Only one participant had previoustraining in drug-allergy recognition and management.

There appeared to be prevalent misconceptions that anaphylactic reactions to BPG were common and were increased.in individuals who were fasting or otherwise weak. Mostprogramme participants were not aware that prior tolerance of other forms of penicillin (such as oral penicillin VK or intramuscular penicillin G) might have a bearing on thesubsequent risk of anaphylaxis to BPG. A small number ofparticipants inquired whether penicillin allergy testing would be necessary before BPG administration.

## Educational session

Informed by observations in the focus group, the educational component of the workshop covered the following topics: streptococcal pharyngitis and its relationship to RF and RHD; the role of penicillin in primary and secondary prevention; review of the various forms of penicillin, including BPG, penicillin VKand penicillin G; use of penicillin in previously documented RHDcontrol programmes; the nature and likelihood of possible adversereactions to penicillin (including IgE-mediated type I allergicreactions and other non-allergic adverse reactions); and how torecognise and intervene in acute anaphylaxis. The educationalsession also reviewed evidence that supported the lack of need toconduct penicillin allergy testing (often simply called ‘skin testing’locally) before BPG administration to a patient in whom there wasno prior history of adverse reaction to penicillin.

**Fig. 1.**
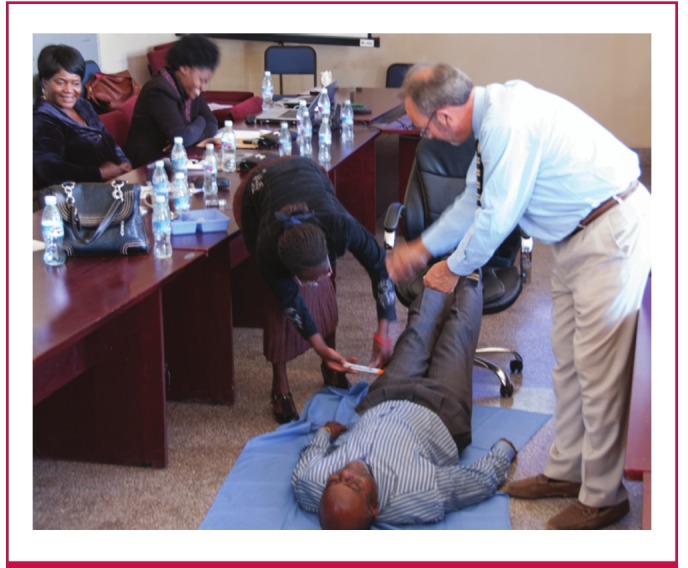
A skills-building, role-playing exercise was conducted at the Lusaka workshop to help nurses and doctors build confidence in their ability to successfully recognise and manage medication-induced allergy. Placing the patient on the back and elevating the lower extremities is recommended for management of anaphylaxis, in addition to the immediate administration of adrenaline. 31

Following the didactic programme, a hands-on, role-playing exercise was undertaken to teach recognition and management of acute anaphylaxis in a simulated patient (Fig. 1), based on algorithms developed by the World Allergy Organisation.^29^ Skills imparted included placing the patient in the supine position with the legs elevated, proper assessment of the patient’s airway, correct administration of intramuscular epinephrine, and determination of the potential need for additional medications such as antihistamines and bronchodilators.

Educational activities were evaluated by pre- and posttesting of knowledge and skills. All participants demonstratedsignificantly improved anaphylaxis management skills, and in ananonymous post-course evaluation, every participant reportedthat their clinical practice would change as a result of the course.

## Workshop learnings

Important lessons learned from the initial educational workshop guided future programme activities. First, it was clear that health workers in Zambia had had misconceptions about thetrue frequency of severe penicillin allergic reactions. Second,health workers received scant, if any, training in drug-allergyrecognition and management; therefore there was a need for programmes to improve health workers’ confidence in managing patients with drug allergy. Third, health workers were unclearabout the precise indications and dosing for administering BPG,and were eager for opportunities to improve their diagnosticand treatment skills. These were each felt to be remediable contributory factors to the suboptimal use of BPG for primary and secondary prevention of RHD in Zambia.

## Design and deployment of subsequent tailored interventions

 A core activity of the BeatRHD Zambia initiative is to work to helpstrengthen the Zambian health system in order that services forprimary and secondary prevention of RHD are reliably delivered.To achieve this, an RHD control programme was developed forimplementation in individual health facilities, which includesan introductory on-site training workshop, dissemination ofeducational materials for staff and patients, ongoing supportivesupervisory visits by UTH staff, and assessment of BPG stocks.Largely as a result of the lessons learned in the initial workshopdescribed above, four main interventions were incorporated into the RHD control programme in Zambia.

## Creation of durable and accessible educational materials

**Fig. 2.**
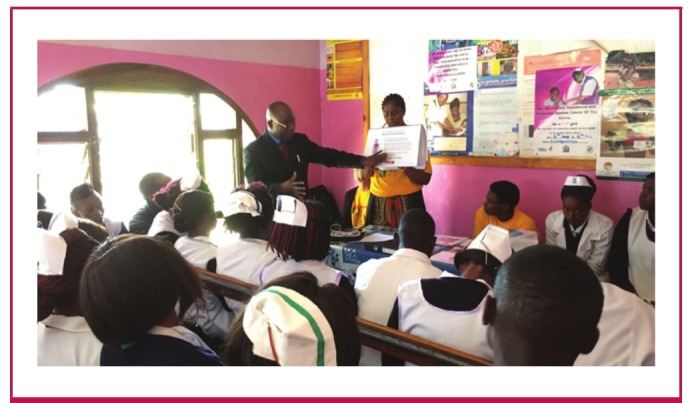
The BeatRHD Zambia team conducts an on-site introductory workshop during enrollment of new healthcentres into the RHD control programme. The educationalsession was flipchart based and included allergy training as a core component.

A user-friendly allergy-focused educational module, based on presentations delivered in the original workshop, was developed into a laminated paper flipchart format for subsequent teachingand reference in the field without need for electronic audiovisual support (Fig. 2). The flipchart reviews the topic of drug allergy; how to recognise and manage a severe allergic reaction; how the allergy kit is used (see below for description of allergy kit); and which medicines are indicated for patients with a known allergy to penicillin. A professionally produced video recording of the allergy module content was also developed for free electronic distribution, and a link to the video file was posted to the PASCAR website for educational purposes.^30^

## Compilation and provision of penicillin allergy kits

**Fig. 3.**
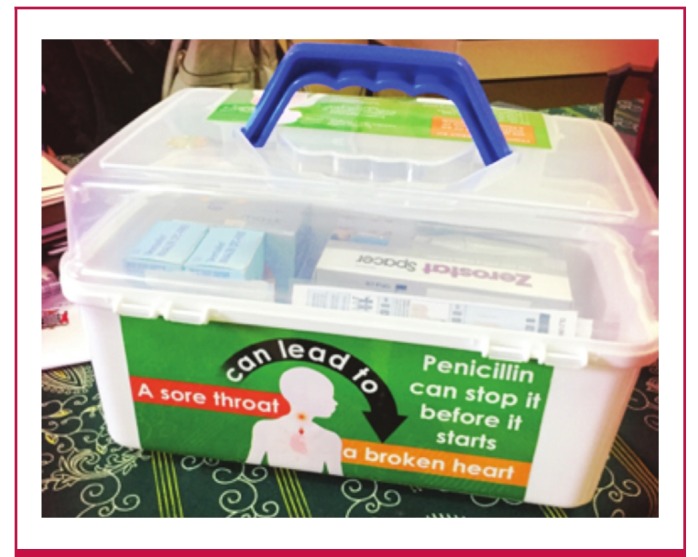
A specially designed, bundled allergy kit was assembled and distributed to each health centre enrolled in the RHD control programme. The kit containskey medicines and other materials, including pictorialinstructions, needed to initiate management of asevere drug-allergy event.

Every health centre that is enrolled in the RHD control programme is provided with a bundled ‘penicillin-allergy kit’ that contains the key materials needed to initiate managementof a penicillin-induced allergic reaction (Fig. 3). The allergy kit was conceived to be an additional mechanism that complements training, to help physically prepare health workers to manage drug allergy, to help build their confidence so that they could successfully manage an allergic event, and to ultimately reduce barriers to the administration of injectable penicillin.

The allergy kit contains a set of medications consistent with World Allergy Organisation guidelines for treating drug allergy,^29^ including injectable epinephrine with a sterile syringe andalcohol wipes; an oral non-sedating antihistamine; a short-acting beta-agonist bronchodilator inhaler; and oral prednisone tablets. These kits also include concise instructions for emergency stepsto be taken in the event of a serious allergic reaction, a photocopyof figures from the World Allergy Organisation guidelines, a data sheet to record clinical events, a pen to complete the data sheet, and a patient handout. The components are packaged togetherin a locally procured, conspicuously labelled plastic box thatwas designed for ready availability and ease of transport. The medicines in the allergy kit are clearly displayed and labelled to facilitate quick and proper use.

## Ongoing supportive supervision in clinics

Nurses from UTH provide on-site supportive supervision once or twice monthly to each clinic enrolled in the RHD controlprogramme. These visits have been determined to be necessary in order to provide regular refresher education and training relating to drug allergy and other aspects of the RHD controlprogramme (for example, primary and secondary prevention);to answer questions and help solve problems that invariablyarise at the point of care; to check and re-stock the allergy kit asnecessary; and to confirm that each health centre’s pharmacy hasan adequate stock of penicillin, including BPG.

## Assessment of BPG availability

Through the RHD control programme, free penicillin treatment is offered to patients for primary and secondary prevention of RHD. To help ensure availability of high-quality medicine,in-country stocks of penicillin were augmented by a productgrant of 25 000 doses from Sandoz, in accordance with World Health Organisation guidelines for medicine donations.^31^ The product grant was a supplement; we found that BPG procured through normal government processes was virtually always also available in enrolled clinics.

Training followed by supportive and mentorship visits have also been commenced in provinces outside Lusaka, with thefirst one being in Choma (Southern Province), where ongoingsupportive supervision in clinics and assessment of BPGavailability will be replicated.

## Initial outcomes

Baseline information obtained from the initial workshop indicated an extremely low (and perhaps even zero) rate of usage of BPG for primary and secondary prevention of RHD amonghealth workers at UTH and Lusaka area government healthfacilities. Now, two years later, we have observed substantialchanges in the pattern of BPG usage as a result of the programme’s interventions.

We conducted structured interviews with 18 nurses, clinical officers and pharmacists in seven clinics that had been enrolled in the RHD control programme for four to six months. Ninety per cent of respondents had administered injectable penicillin since the training, and most of them reported that they had administered the medicine on many occasions. Six of the 18participants reported that using injectable penicillin to treatpharyngitis was a new practice for them, which they had learned as a result of the programme. None of the health workers thought it was too much work to administer injectablepenicillin compared with pills. Only one nurse had apprehensionabout giving injectable penicillin, and she requested from theprogramme nurses more training on allergy recognition and management; all other health workers reported that they felt comfortable recognising and managing penicillin allergy as a result of the knowledge and skills gained in the training.

All heath workers interviewed believed that patients actuallypreferred injections to pills due to the perception that it was amore effective treatment. Many of the respondents also reportedthat they preferred the 1.2 million IU formulation to the 2.4million IU formulation, since it was easier to dose in children. While the number of participants in this pilot evaluation was small, we believe that it provides an early signal on the impactof the programme.

To date, 21 government health facilities in Lusaka have been enrolled in the RHD control programme and records indicatethat more than 9 000 doses of BPG have been administered since the programme started, the majority of which was used for primary prevention of RHD (the incidence of pharyngitiswas much higher than the number of patients with RHD whorequired secondary prophylaxis). Penicillin-allergy skin testingprior to BPG administration was not routinely undertaken. Nocase of anaphylaxis has been recorded. Further scale-up of theRHD control programme in Lusaka Province is underway, as is expansion to the Southern Province. Extension to additional provinces is anticipated during 2017.

## Discussion

Rheumatic fever and RHD are preventable and potentially eradicable conditions that still account for significant morbidity and mortality rates in Zambia, other countries in SSA and other underdeveloped areas of the world. Low rates of penicillin use,including BPG, in appropriate clinical circumstances are likelyto be a factor accounting for the continuing high prevalence ofthese diseases.^18^

From our experience, included among the important underlying drivers that contribute to low rates of BPG usageare a lack of appropriate knowledge regarding the confirmedbenefits to be derived from its use and the fear of potential adverse events, including allergic reactions. Similar observationshave also been made in other regions of the world where RHD isendemic.^32^ That fear of penicillin allergy emerged as a significant barrier to BPG use, which came as somewhat of a surprise, since apprehension towards drug allergy has not been commonlydescribed among health workers in SSA, and scant literatureexists on adverse penicillin reactions in population-based studies of RF and RHD.

A multinational study in 1991 that included 32 430 BPG injections in 1 790 patients estimated the risk of anaphylaxis to be exceedingly low, at approximately one in 10 000 injections,^33^ and a 2014 retrospective study of BPG treatment in RF in Turkey found confirmed allergy in one of 535 patients (0.18% of 17 641 injections) but documented no anaphylactic reactions.^34^ Three fatalities that were temporally related to BPG injection were reported from Zimbabwe more than 15 years ago, although clinical details were not well described and so it is not clear that drug allergy played a role.^35^

We document here that relatively simple interventions, including appropriate education of healthcare personnel, together with confidence building around the recognition and management of allergic drug reactions, followed by a number of low-cost ongoing supportive measures have the potential to significantly improve rates of BPG usage in the primary and secondary prevention of RHD in a low-resource setting.

Our early data also suggest that there is no need to perform routine penicillin-allergy testing prior to BPG administration in patients without a prior history of adverse reactions to penicillin.This position is supported by several large published studies that evaluated interventions with BPG in RHD patients, wherelarge numbers of BPG injections were administered withoutprior penicillin-allergy testing,^33^,^34^ and reported the incidence of adverse reactions, including anaphylaxis, was exceedingly low.^36^

A key action recommended in the plan to eradicate RHD inAfrica includes appropriate training of health workers to safelyand effectively deliver BPG.^22^ The preliminary experience in Zambia suggests that appropriate educational interventions in the setting of drug availability and ready access to medications to treat anaphylaxis can positively impact on rates of BPG usage. Future work will involve the exploration of innovative ways to scale up the RHD control programme, such as the use of electronic training modules, and determination of the impact of these types of interventions on health outcomes, including the incidence of RF and RHD.

## Conclusion

A multi-faceted effort to combat RHD in Zambia included, as a core component, a novel programme to demystify concerns and dispel fears about safe administration of BPG. It appears that this approach contributed to increases in the rate of BPG use for primary and secondary prevention of RHD in government health facilities, according to national guidelines. Lessons from this experience may be applicable to other countries where RHD is endemic.
